# ABrainVis: an android brain image visualization tool

**DOI:** 10.1186/s12938-021-00909-0

**Published:** 2021-07-29

**Authors:** Ignacio Osorio, Miguel Guevara, Danilo Bonometti, Diego Carrasco, Maxime Descoteaux, Cyril Poupon, Jean-François Mangin, Cecilia Hernández, Pamela Guevara

**Affiliations:** 1 Department of Computer Sciences, Universidad de Concepción, Concepción, Chile; 2 Department of Electrical Engineering, Universidad de Concepción, Concepción, Chile; 3 Center for Biotechnology and Bioengineering (CeBiB), Santiago, Chile; 4 Université Paris-Saclay, CEA, CNRS, Neurospin, BAOBAB, Gif-sur-Yvette, France; 5 Sherbrooke Connectivity Imaging Lab (SCIL), Computer Science Department, Université de Sherbrooke, Sherbrooke, Canada

**Keywords:** Mobile visualization, 3D rendering, Brain imaging

## Abstract

**Background:**

The visualization and analysis of brain data such as white matter diffusion tractography and magnetic resonance imaging (MRI) volumes is commonly used by neuro-specialist and researchers to help the understanding of brain structure, functionality and connectivity. As mobile devices are widely used among users and their technology shows a continuous improvement in performance, different types of applications have been designed to help users in different work areas.

**Results:**

We present, ABrainVis, an Android mobile tool that allows users to visualize different types of brain images, such as white matter diffusion tractographies, represented as fibers in 3D, segmented fiber bundles, MRI 3D images as rendered volumes and slices, and meshes. The tool enables users to choose and combine different types of brain imaging data to provide visual anatomical context for specific visualization needs. ABrainVis provides high performance over a wide range of Android devices, including tablets and cell phones using medium and large tractography datasets. Interesting visualizations including brain tumors and arteries, along with fiber, are given as examples of case studies using ABrainVis.

**Conclusions:**

The functionality, flexibility and performance of ABrainVis tool introduce an improvement in user experience enabling neurophysicians and neuroscientists fast visualization of large tractography datasets, as well as the ability to incorporate other brain imaging data such as MRI volumes and meshes, adding anatomical contextual information.

**Supplementary Information:**

The online version contains supplementary material available at 10.1186/s12938-021-00909-0.

## Background

**Fig. 1:**
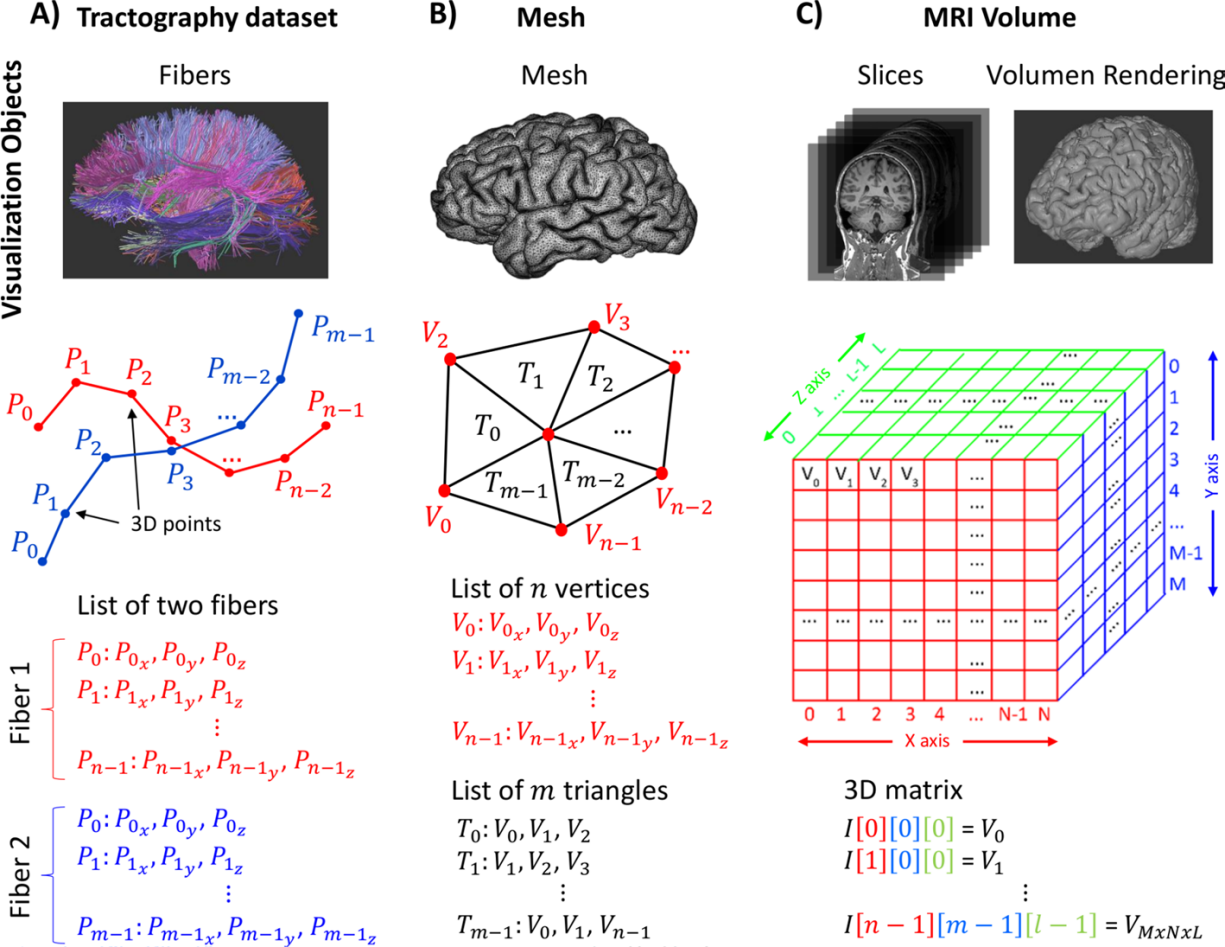
Main data formats supported by ABrainVis. **A** Tractography dataset, composed of fibers or streamlines. Each fiber is represented by a 3D polyline consisting of a set of 3D points. Other information may be included, such as fiber bundle names or a spatial transformation, not represented here. **B** Mesh file, composed of a list of 3D vertices (3D points), and a list of triangles, where each triangle is defined by the indices of the three vertices that compose it. Other information can be included, such as vertex normals (not represented here). **C** MRI volume, composed of a 3D matrix of voxels (volume elements), where each voxel is associated with an intensity. The file also contains other information, such as voxel dimensions, included on a header (not represented here). Both tractography datasets and meshes have associated only one display object each, while MRI volumes can be displayed as slices or volume rendering

Neuroscientists and neurophysicians typically use visual inspection of brain imaging as a way of understanding the structure and extracting useful information of the brain, as well as to perform quality control. However, high-quality brain images usually require large high dimension datasets and developing fast, interactive and flexible visualization tools is a challenging task. For instance, tractography datasets are constructed based on the diffusion local models from diffusion magnetic resonance imaging (dMRI) [1], that measures the 3D motion of white matter molecules in the brain. Tractography data, representing the main 3D white matter fiber pathways, are commonly composed of a set of fibers or streamlines, where each fiber is a discrete 3D line formed by a set of 3D data points, also known as 3D polyline. See Fig. 1A for a schematics of tractography data representation. With the development of different diffusion models [2, 3], currently tractography datasets can have over one hundred thousand of fibers. In addition, there exist software tools to cluster fibers with similar shapes and lengths [4–6] and segment bundles based on a fiber bundles atlas [7–9]. These tools enable the visualization of such structures and can be of interest for analyzing their potential anatomical meaning.

Other types of brain imaging, such as MRI images and meshes are also of great interest for visualization (see Fig. 1B, C for a schematics of mesh and MRI volume representation). These types of data are relevant for providing a more complete view of the anatomical context. For instance, visualizing different fiber bundles connecting different brain regions can be better understood when visualized in combination with MRI volumes and slices, to see clearly which other organs can be close. The flexibility of a mobile visualization tool, able to display tractography datasets, supporting the inclusion of other brain images can be of special interest. For example, a neurophysician might need to visualize a brain tumor in the context of MRI volume and fiber bundles in the neighbor region, to extract more information. Being able to visualize different brain images in a combined way is also challenging because in addition to data size, the method must deal with different formats and provide different visualization operations and interactions, smoothly and efficiently.

There exist several applications that enable image visualization and analysis for brain research studies. Most of these tools work on general purpose computer systems [10, 11] or are web-based [12–15]. As the mobile industry, including tablets and cell phones devices, keeps growing in the number of users and improving in performance, its use in different working areas is becoming increasingly common. Several mobile visualization tools for neurophysicians and researchers have been proposed. Some of these applications have general educational purposes or seek to instruct their users regarding more specific areas. These applications allow the user to navigate through different preset data, for instance the free mobile version of BrainTutor [16], that includes brain cortex information from a 3D object; Atlas of MRI Brain Anatomy [17] and Brain MRI atlas [18], both inform about the brain structures from 2D MRI slices; NeuroNavigator [19] that offers the 3D visualization of structures from the brain cortex, blood vessels, functional activation and fiber pathways; MRI Viewer [20] that includes MRI images of neck, chest and pelvis; CT Scan Cross Sectional Anatomy for Imaging Pros [21] that shows 2D slices of computer tomography (CT) images from the body, complemented with educational drawings. Other applications target more specific areas for instructing medical trainees, going from a general learning as the radiology of the whole body (Radiological Anatomy For FRCR1 [22]), or the typical artifacts and variants in different imaging modalities (Imaging Brain, Skull and Craniocervical Vasculature [23]), to more specific ones as brain MRI atlases (NeuroSlice, [24]) or the myelination changes in the brain (Myelination Brain, [25]). Other applications of this type aim to more practical objectives as the aid in surgical scenarios. For instance the work of Dogan et al. [26] proposes an application that allows the user to input their own data where brain lesions are identified, and superpose it over the actual patient’s head by using the device camera. Another work, presented by Rojas et al. [27], proposes a tool for the visualization of functional connectivity networks and the relative positions of EEG electrodes from preset data, in order to facilitate this kind of exams.

Other available applications offer the user the possibility of visualizing their own data as mRay [28] (MRI data) or IMAIOS Dicom Viewer [29] (ultrasound, scanner, MRI, PET, etc.). However, these tools only provide 2D visualizations of the 3D volume data. A summary of all these applications can be seen in Additional file 1: Table S1.

Furthermore, there exist some high-performance 3D objects viewers available for mobile devices (3D Model Viewer-OBJ/STL/DAE [30], 3D Model Viewer [31]), but these are not focused on medical imaging data. Moreover they do not offer a direct 3D rendering from images.

As mentioned, most existing applications allow users only to visualize their own preset data, which limits their uses. Although some of the applications allow the user to load their own data, their functionalities are reduced to few imaging modalities or visualization modes. Taking this into account, in this work we propose a visualization tool for Android mobile devices that enables the visualization of user’s different types of brain imaging data. This work is an extension of our previously presented application  [32], which proposed an efficient Android tool for large tractography datasets, enabling fast visualization of large number of fibers, and includes interactive operations using the graphic OpenGL pipeline framework. In this new release we considered the visualization of a wider range of data types, including the visualization of 3D medical images and meshes, that can be displayed independently or combining different imaging data formats. The supported types are generics, allowing the users to include different data, such as brain tumors and arteries, in the form of 3D images or meshes, not being exclusive for brain imaging, which allows researches to improve the analysis using different case studies.

## Implementation

ABrainVis supports three main data formats: tractography datasets, meshes and MRI volumes. Figure 1 presents the main representation of these data formats. Both tractography datasets and meshes have associated only one display object each, while MRI volumes can be displayed by using two different display objects. The first is a *slice* object, which is a 2D image of the volume that is aligned with the volume axes. The second is a 3D volume rendering object, which performs a direct rendering of the three-dimensional image data. Tractography datasets are composed of fibers or streamlines, where each fiber is represented by a 3D polyline consisting of a set of 3D points. Depending on the specific file format, additional information may be included, such as fiber bundle names (*.bundles* format) or a spatial transformation (*.trk* format). Mesh files are more generic data types. These are composed of a list of 3D vertices (3D points), and a list of polygons, commonly triangles, where each triangle is defined by the indices of its vertices. Another type of information, such as the vertex normals, can be included. An MRI volume is represented with a 3D matrix of volume elements (voxels) distributed on a 3D regular grid, where each voxel is associated with an intensity. Additional information, such as voxel dimensions, can be included in the input file header.

**Fig. 2:**
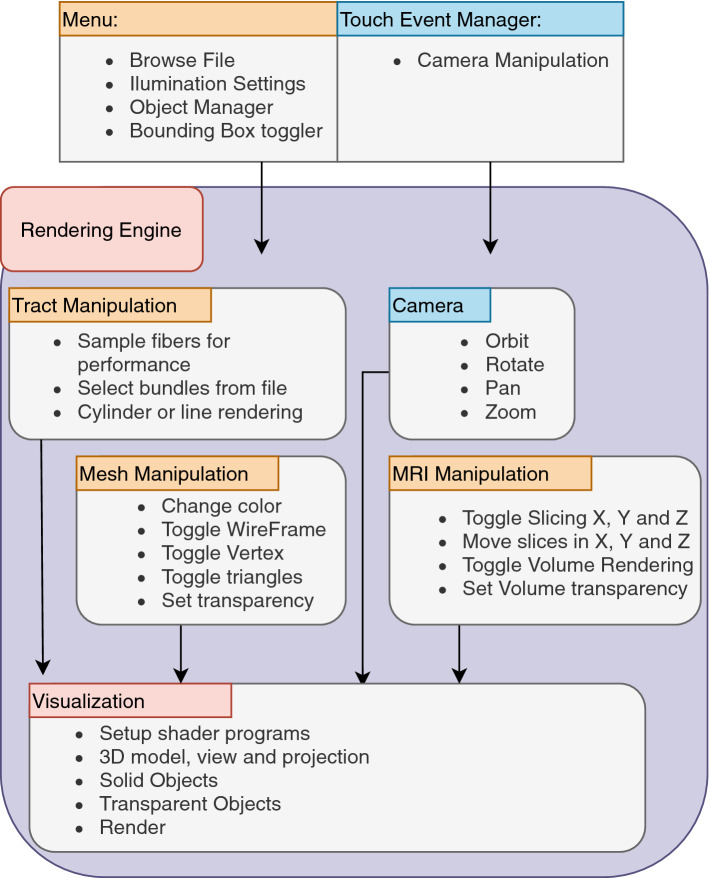
ABrainVis software components and interactions. The user’s interface component (GUI) consists of a Menu and an Event Manager module. This component connects with the rendering engine which contains modules to manipulate the camera, visualization, and the supported visualization objects (tractography datasets, meshes and MRI volumes)

ABrainVis provides different functionalities aiming to provide a fast visualization and an intuitive user interface. In order to provide a fast 3D rendering, the tool uses the OpenGL 3D graphic engine. The main components of the visualization tool are the Graphic User Interface (GUI) and the Rendering Engine, which are displayed in Fig. 2. As shown, the user interface component consists of a Menu and an Event Manager module. This component allows users to load files, define visualization settings and interactively adjust object views.

The GUI component communicates with the rendering engine which uses OpenGL ES (OpenGL for Embedded Systems) [33] pipeline visualization framework. The rendering engine has modules to manipulate the camera, visualization, and the supported visualization objects (tractography datasets, meshes and MRI volumes). The ABrainVis graphic component uses extensible OpenGL shaders, which exploit parallelism for processing graphic operations using OpenGL data specialized objects, such as VBO (Vertex Buffer Object) and EBO (Element Buffer Object), for vertex and index high-performance processing. A VBO enables the processing of methods to upload the vertex data properties, such as positions, normal vectors, and color, while an EBO stores indices that OpenGL uses to decide which vertices to draw. The main features of the rendering engine modules are described below.

### Fiber tract manipulation

This module processes all tasks related to fiber tractography operations, such as loading fiber bundles from files, choosing fiber visual representation, bundle selection and fiber sampling. Tractography files contain the 3D coordinates for each point of each fiber in the dataset. The number of points can be different for each fiber. The application supports two formats: TrackVis format (files with extension *.trk*) [34] and Bundles format (two files with extensions *.bundles* and *.bundlesdata*) [35]. Bundles format supports the use of labels, hence tractography datasets can contain segmented bundles or clusters, identified with a name.

#### Data objects

Tractography datasets are loaded and represented in VBO buffers. These buffers are used to perform required processing for the tractography dataset, without the need of operating with immediate rendering. In addition, this module uses the OpenGL EBO buffers to store the vertex indices, which are used for selecting specific vertices to render.

The tractography visualization also allows a user to represent fibers visually as lines or cylinders. Both visualization modes support illumination. In the case of line display, the normal is emulated using the local fiber direction, where for each point it is calculated as the direction of the next fiber segment. The cylinder representation provides a more realistic appearance, improving the quality of the visualization, but decreases the rendering performance.

#### Data sampling

The visualization of complete large tractography datasets might be demanding in terms of memory usage and rendering time. The current version of ABrainVis stores the complete tractography dataset in VBO buffers and, since visualizing all fibers can consume too much memory and processing time for the device, the tool supports fiber sampling. Note that the resource usage requirement is also true for general purpose computers or laptops. The sampling can be selected as a percentage of fibers, from 1% to 100%.

#### Fiber bundle selection

This operation allows a user to select fiber bundles from a label list given with the input data for Bundles format. The bundle labels are not part of ABrainVis processing, they need to be produced by a previous step such as applying a clustering or segmentation method. In order the keep the visualization processing fast, ABrainVis creates a new EBO for each bundle to be rendered. As the number of bundles, as well as some bundles in a file can be large, sampling is also an alternative that the user can choose to improve visualization time and reduce data occlusion. In addition, different colors are randomly selected for each bundle.

### MRI manipulation

The volume and slice rendering are based on 3D images in NIfTI data format [36], including the file extensions *.nii* or *.nii.gz*. This module reads the data into a 3D matrix and, if provided, a transformation matrix. The 3D matrices are stored as textures in OpenGL objects, which can be processed using specialized methods for each rendering mode. The base technique used for both renderings is described by Hadwiger et al. [37], and are briefly outlined below.

#### Slice rendering

The slice manipulation allows a user to visualize 2D cross-sections of the volume. The tool enables the visualization of one slice for each one of the three planes, according to the “*X*”, “*Y*”, and “*Z*” coordinates of the image. These slices will correspond to the axial, sagittal and coronal planes of the head, depending on the orientation of the image. A slider allows the user to modify the variables describing the slice number for each plane. Then, a volume slice is calculated on the GPU using the OpenGL pipeline. The implementation enables to extend the module for creating slices with planes that are not perpendicular to the main three axes. The rendering is performed, without interpolation over the plane, by fetching the intensity values from the 3D texture. The slice is colored according to a simple injective linear function that maps the values $f:{\mathrm{3DTexture}} \rightarrow \mathcal {R}$.

#### 3D volume rendering

The volume rendering uses the same techniques as the slice rendering explained above. The rendering process draws a group of slices, covering the whole volume for the plane perpendicular to the viewing vector. The plane is drawn using a number of slices ($n_{\mathrm{s}}$) over the volume, moving along the viewing direction by a uniform spacing. The number of slices to be drawn is determined in terms of the number of slices of the volume in each axis ($n_x$, $n_y$ and $n_z$) and a sampling factor (sf). By default sf is set to 0.2 (see Eq. 1):

1 $$\begin{aligned} n_{\mathrm{s}} = {\mathrm{sf}} \times \sqrt{n_x^2 + n_y^2 + n_z^2}. \end{aligned}$$

To define the intensities to be considered, the Otsu thresholding algorithm [38] is used to select a proper threshold for the volume. The lighting effect is calculated using the gradient estimation and Phong illumination algorithm [37, 39]. A good performance is achieved thanks to the modern OpenGL functionalities, such as *instancing drawing*, for fast rendering of multiples planes. Also, *shader processing* is used for the lighting algorithm, which is executed on the GPU using the OpenGL framework. All important values are programmed to be easily modified and added to the GUI if necessary. The tool allows the user to modify different parameters of the visualization of slice and volume rendering through its graphical user interface.

### Mesh manipulation

The mesh processing supports GIfTI (files with extension *.gii*) [40], and Mesh (two files with extensions *.mesh* and *.mesh.minf*) [41] formats. All meshes contain graphical surface-based data including 3D vertex coordinates, triangle vertex indices (geometry information), and optionally, the normal vector for each vertex. If normal vectors are not included in the input data, the tool computes them. To produce proper transparent objects, the mesh is drawn from back to front, using a sorting algorithm and the user viewing vector (*eye* perspective). The wireframe can also easily be activated through the GUI. Different colors can be selected for triangles and wireframe. The effects are obtained by modifying the OpenGL options.

### Camera

**Fig. 3:**
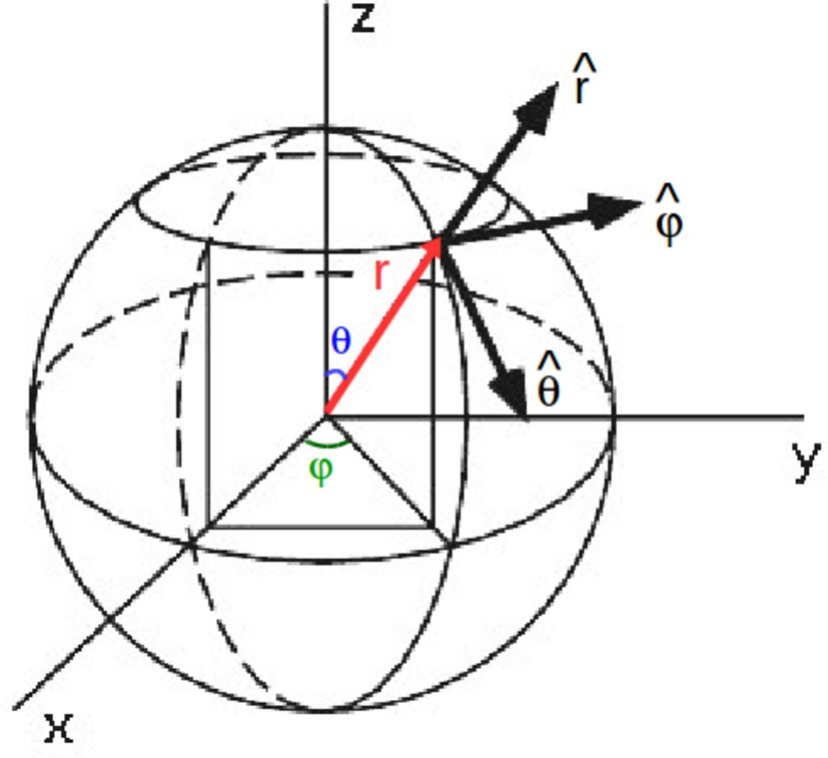
Camera coordinates internal representation. The center of the sphere, located at the origin, is the point where the camera looks, *r* is the position of the camera. The angles $\theta$ and $\varphi$ are stored as a rotation quaternion to facilitate camera manipulation. The three vectors are spherical coordinate representations of the plane of view

The camera module manages the object position, focus and orientation. The user interface interacts with this module through the event manager, by providing the operation required by the user such as orbit, rotate, pan and zoom. These operations modify the axis angles, camera origin coordinates and radius of the visualization matrix. To support 3D objects, the camera module uses spherical coordinates as shown in Fig. 3. As the users are able to modify the objects view by touching the device screen, this module supports the following interaction operations. *Orbiting* by swiping the screen with one finger, *rotating* by pinching the screen with two fingers, *zooming* by pinching the screen with two fingers, and *panning*, by swiping the screen with two or more fingers.

The camera module communicates with the visualization module, which is responsible for processing and displaying the actual graphic objects on the screen.

### Visualization

This module performs the object rendering. It contains the programmable shaders, the visualization matrix and illumination constants.

Shaders represent small programs that run on the GPU, controlling small parts of the OpenGL pipeline. They normally take an input and transforms it into an output to the next stage. There are three main types of shaders, the Vertex Shader (VS), the Fragment Shader (FS) and the Geometry Shader (GS). The VS allows to deal with the geometry information of the vertices, the FS generates the rendered pixels, and the GS is optional and enables the extension or reduction of the geometry and is executed after the VS. As shaders are the parallel processing functions executed on the GPU of the device, they are defined according to the object type required to display. All shaders use the VBO and EBO buffers, where the EBO buffer indicates to OpenGL which objects, residing as vertices in VBO buffers, to render. The vertex shader is responsible for drawing vertices, while the line shader and cylinder (extension) shader draw lines and cylinders, correspondingly. For fast tract manipulation, ABrainVis uses OpenGL ES mode called *primitive restart* to draw all fibers in only one function call.

The visualization module also implements the illumination 3D model using the Phong algorithm [39]. The color displayed at each pixel depends on the color of the object, given by the material reflection constants, and the effect of the shading, defined by the lighting parameters. The algorithm computes the illumination (*I*) at each surface point as a function of the ambient ($L_{\mathrm{a}}$), diffuse ($L_{\mathrm{d}}$), and specular ($L_{\mathrm{s}}$) light components, material reflection constants (ambient $K_{\mathrm{a}}$, diffuse $K_{\mathrm{d}}$ and specular $K_{\mathrm{s}}$), the view vector (${\hat{\varvec{v}}}$), the light reflection vector (${\hat{\varvec{r}}}$), the plane normal vector (${\hat{\varvec{n}}}$), the direction vector from the point toward the light source (${\hat{\varvec{l}}}$), and the shininess coefficient (*f*). The illumination computation is shown in Eq. (2), where all the constants ($L_{\mathrm{a}},L_{\mathrm{d}},L_{\mathrm{s}},K_{\mathrm{a}},K_{\mathrm{d}},K_{\mathrm{s}}$) are in the range [0, 1] and the vectors are normalized. This algorithm is used in the vertex shader.

2 $$\begin{aligned} I = L_{\mathrm{a}}K_{\mathrm{a}} + L_{\mathrm{d}}K_{\mathrm{d}}({\hat{\varvec{s}}}\cdot {\hat{\varvec{n}}}) + L_{\mathrm{s}}K_{\mathrm{s}}({\hat{\varvec{r}}}\cdot {\hat{\varvec{v}}})^f. \end{aligned}$$

**Fig. 4:**
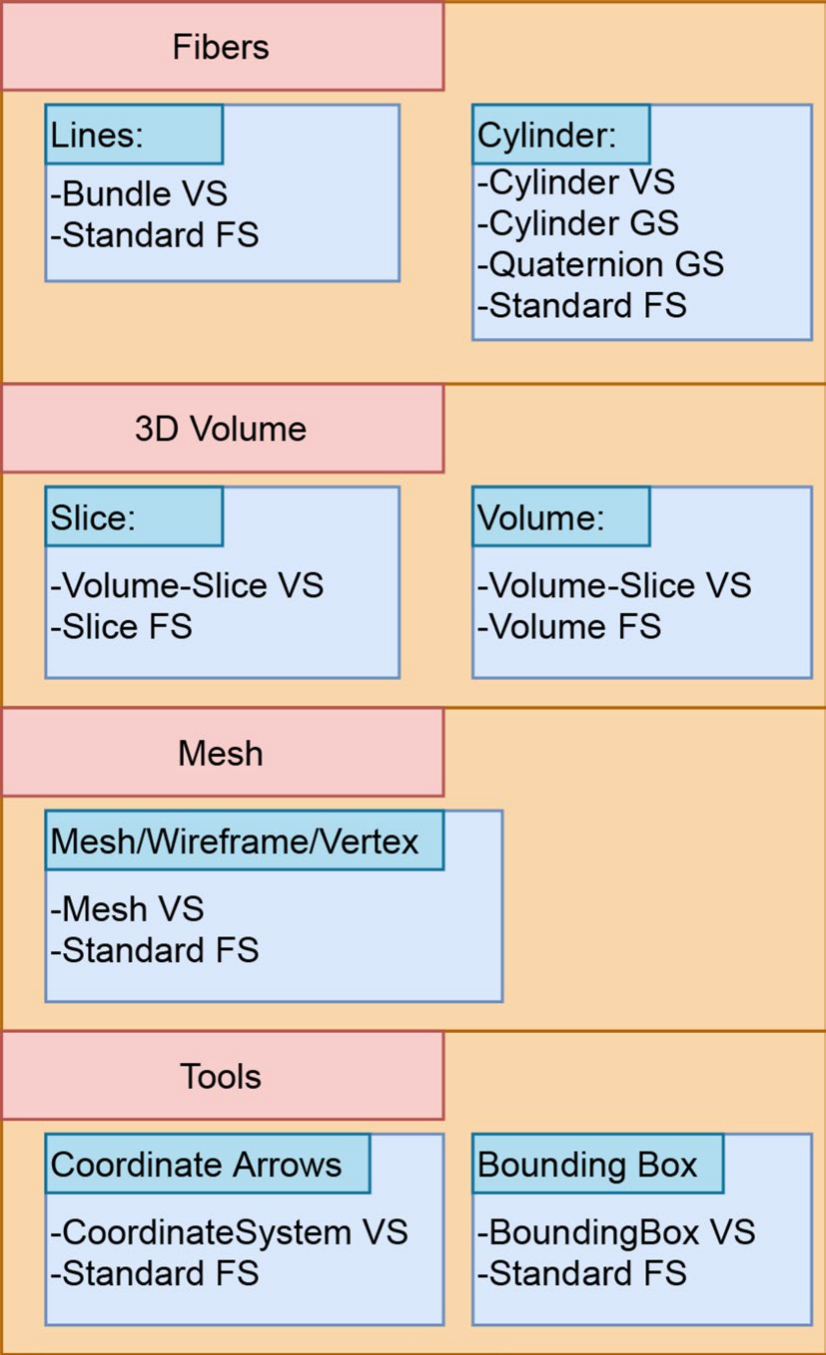
A diagram showing the different types of data that the application can display, along with the supported visualization modes and the shaders used to render them.* VS* vertex shader,* FS* fragment shader,* GS* geometry shader

The rendering effects are generated using the combination of these shaders, described in Table 1. For each type of data, different shaders are needed, which are summarized in Fig. 4. For the *Tract visualization*, we use a special vertex shader for adding lighting to the fibers. When using the cylinder render option, the vertex shader passes the vertex information to the next pipeline stage, where a *geometry shader* is used to extend lines to cylinders, while adding the lighting.

**Table 1:** Shaders used for visualization

Type	Name	Description
Vertex shader	Bounding box	Model and view matrix applied to lines without lighting effect
	Bundle	Model and view matrix applied to tract 3D data with Phong lighting algorithm
	Coordinate system	Model and view applied to volumetrical arrows without lighting effect
	Cylinder	Pass through shader for tract data
	Mesh	Model and view matrix applied to mesh 3D data with Phong lighting algorithm and opacity
	Volume-slice	Model and view matrix applied to vertex from bounding box collided with plane
Geometry shader	Cylinder	Model and view matrix applied to a line-to-cylinder algorithm
	Quaternion	Functions describing quaternion mathematics
Fragment shader	Standard fragment shader	Pass through shader for color data
	MRI slide	Evaluation of pixel color for 3D texture (MRI data)
	MRI volume	Evaluation of pixel color for 3D texture (MRI data) and Phong lighting algorithm using gradient estimation as normal

When rendering slices and volumes, the vertex shader transforms a plane equation into a slice inside the 3D volume. The 3D texture is applied to the resulting slice on the fragment shader, resulting in the slice’s rendering. The volume rendering requires more work to provide a proper perception of depth, achieved by the use of a threshold and a lighting algorithm running on the fragment shader.

The *mesh* has a special vertex shader, which allows the user to modify some variables of interest, such as transparency and color. Similarly, the *tools* object has some very simple dedicated vertex shaders to display the *coordinate arrows* and the *bounding boxes* of the objects.

## Results

This section contains examples with the main functionalities of ABrainVis, describes and discusses the experimental evaluation and results, as well as the datasets used for this purpose.

First, a comparison of the most similar state-of-the-art tools with ABrainVis is shown in Table 2. There is no mobile application with similar features to ABrainVis. The most similar one is *FiberWeb*, which is a web application that allows the user to load tractography data and MRI volumes as slices, but not meshes. It also does not allow color differentiation of different tracts. The most similar mobile applications are *BrainTutor* and *Neuronavigator*, which support viewing meshes, 3D images and fibers, with additional structural information, but they do not allow users to include external data, i.e., only predefined data can be viewed. In addition, neither of these applications supports volume rendering for 3D volumes, nor do they allow users to customize illumination parameters. Although, in some cases, they do offer other functionalities. We include an example of visualization for each one of the three applications in the Additional file 2.

The experimental evaluation considers performance metrics such as frames per seconds (fps) for the tractography datasets using the mobile devices listed in Table 3.

**Table 2:** Comparison between most similar applications

Feature/app	FiberWeb	BrainTutor	NeuroNavigator	ABrainVis
External data	✓	✗	✗	✓
Web/Android/iOS	✓/✗/✗	✗/✓/✓	✗/✓/✗	✗/✓/✗
MRI volume	✓	✓	✓	✓
Mesh	✗	✓	✓	✓
Tractography	✓	✓	✓	✓
Functional connectivity	✗	✓	✓	✗
Zooming	✓	✓	✓	✓
Panning	✓	✓	✗	✓
Rotating	✓	✓	✓	✓
Slice navigation	✗	✓	✓	✓
Transparency	✗	✗	✓	✓
Superimposing	✗	✓	✓	✓
3D rendering	✗	✗	✗	✓
Fiber color	✗	✗	✓	✓(random)
Illumination configuration	✗	✗	✗	✓
Fiber virtual dissect	✓	✗	✓	✗
Fiber tractography	✓	✗	✗	✗
Fiber sampling	✗	✗	✓	✓
Fiber length filter	✗	✗	✓	✗
Structure info	✗	✓	✓	✓

A comparison table between ABrainVis and the most similar applications in the literature. The presence (✓) or absence (✗) of different visualization features is evaluated.

### Datasets

Four datasets were used to display all the different types of data supported by ABrainVis. The same data is used to carry out the performance tests.

#### Dataset I

This is a tractography dataset (*.bundles*) that represents a superficial white matter bundle atlas [42]. It is composed of 100 short association bundles, with a total of 10,622 fibers.

#### Dataset II

This dataset is composed of data of a subject from the ARCHI database [43]. It includes a T1 MRI volume (*.nii.gz*), a head mesh (*.mesh*) and a whole-brain tractography (*.bundles*). The MRI image contains $240 \times 256 \times 160$ slices, with a $1.0 \times 1.0 \times 1.1$ mm resolution. The head mesh is composed of 27,347 vertices conforming 54,722 triangles. The tractography file consists of 36 bundles segmented from a deep white matter bundle atlas [7], using an automatic bundle identification algorithm [8], with a total of 204,052 fibers, resampled with 21 equidistant points.

#### Dataset III

This dataset consists of the data of a patient with a tumor. It has two MRI volumes, a T1 image with $256 \times 256 \times 180$ slices and a resolution of $1.0 \times 1.0 \times 1.0$ mm, and a segmented tumor mask, containing $128 \times 128 \times 74$ slices, with a resolution of $1.8 \times 1.8 \times 1.8$ mm (both in *.nii.gz* format). Also, a whole-brain tractography dataset containing 71,361 fibers is included (*.bundles*). This file is a resampled version of the original file, with only a 4% of the fibers. For this database, we used other tractography dataset, composed of clusters calculated from the original tractography dataset, using the FFClust algorithm [6]. It is composed of all the clusters with more than 200 fibers, consisting of 314 clusters and a total of 117,519 fibers. Neuroscientists usually apply a clustering algorithm on a tractography dataset to perform an exploratory analysis to obtain a general overview of the main structures found in the tractography.

#### Dataset IV

This dataset includes the data of MRI volumes, an arteries mesh, and segmented bundles of a subject. The MRI volume dimensions are $275 \times 332 \times 206$ with a $0.625 \times 0.625 \times 0.625$ mm resolution. The images used are a skull stripped T1 image, and a mask of the segmented arteries from an MRI angiography. Also, an arteries mesh (*.gii*) was calculated from the mask, containing 101,123 vertices and 201,038 triangles. Tractography data are composed of 20 deep white matter bundles in separated *.trk* files that has been segmented using [9], representing a total of 370,613 fibers.

### Main interface

**Fig. 5:**
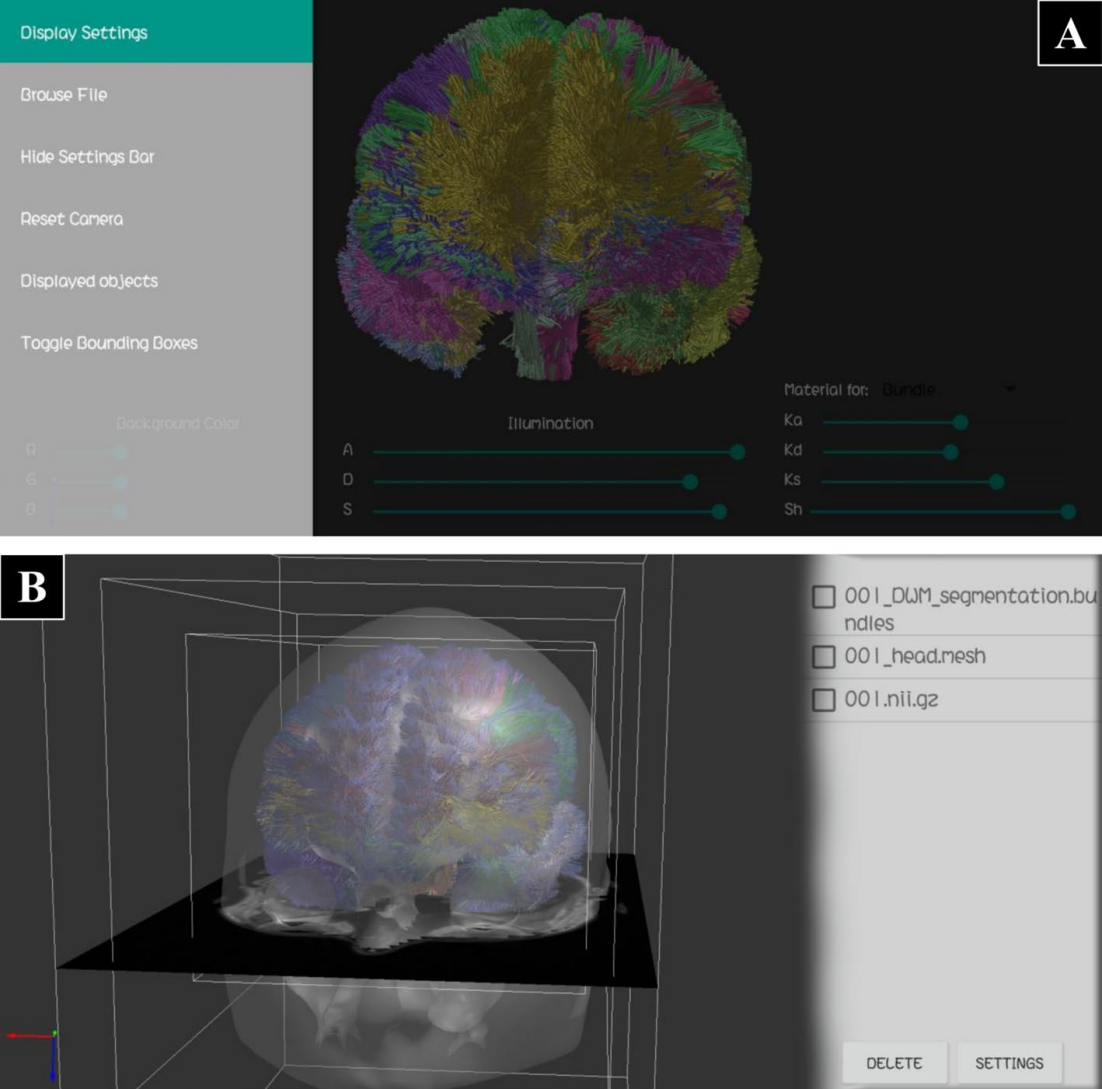
Main components of the application interface. **A** Main screen with the “Settings Bar” visible. When this bar is displayed, the main visualization window and the other setting bars are disabled and obscured. In the main visualization screen a tractography is displayed, as well as the “Display Settings Bar”. **B** Main screen with the “Object Settings Bar” visible, listing the three objects loaded: a tractography dataset, a head mesh and a slice from an MRI volume. Multiple objects can be selected for removing them. Only one object must be selected for displaying its settings options on the bar. In the main visualization screen, the three objects are displayed with their bounding boxes

**Fig. 6:**
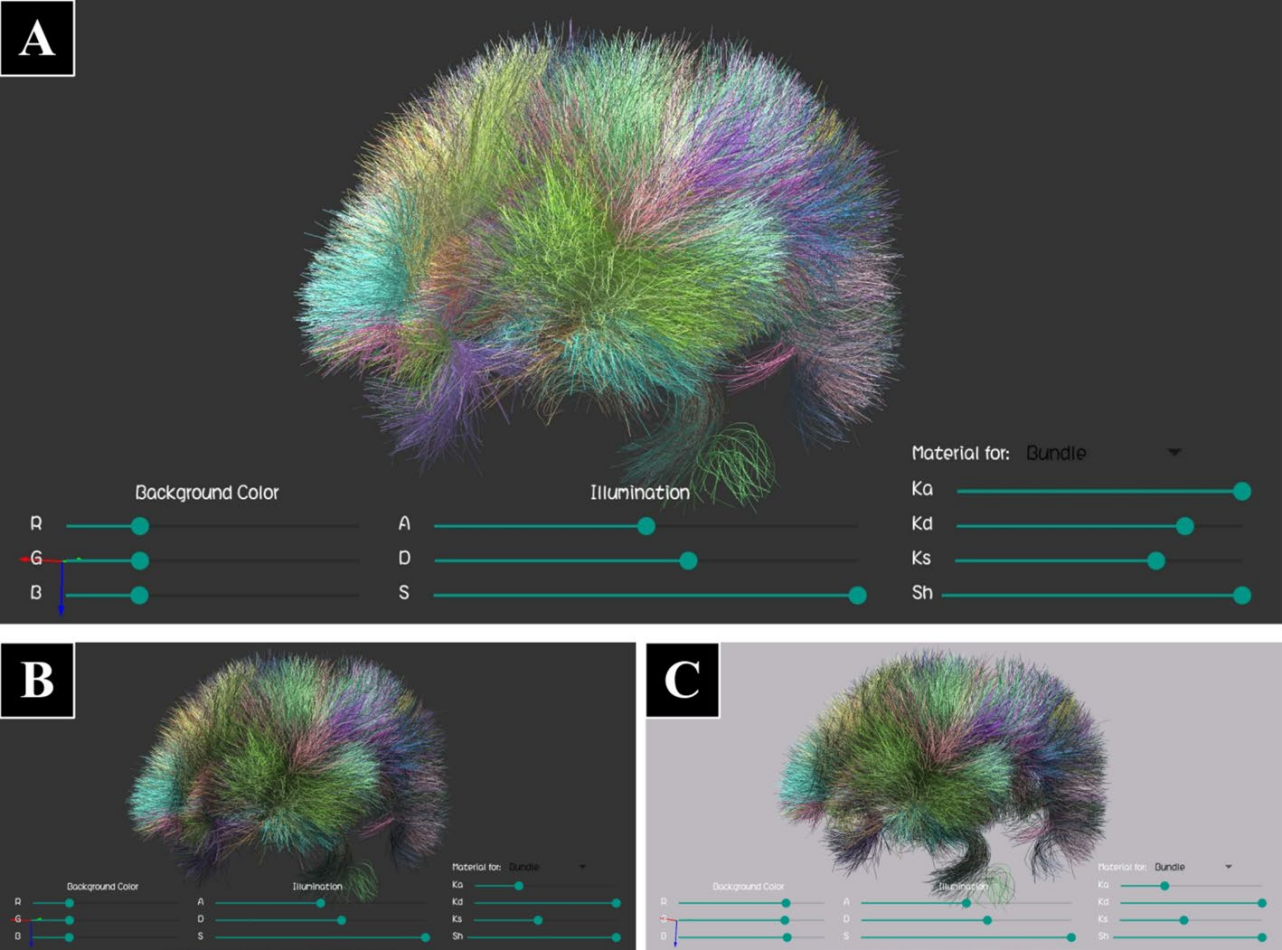
Tractography dataset with a group of labeled bundles representing an atlas of short association bundles (Dataset I), displayed using lines, with different illumination settings. The “Display Settings Bar” is also visible at the bottom of the screen. **A** Default parameters for line display. **B** Changes in the material reflection constants (ambient $K_{\rm a}$, diffuse $K_{\mathrm{d}}$ and specular $K_{\mathrm{s}}$). **C** Additional change in the background color

The main interface is composed of a main display window and a “Settings Bar”, available at the left side of the screen, as shown in Fig. 5A. It has controls to show the “Display Settings Bar”, load files, hide all the settings bars, reset the camera configuration, show the object settings bar (see Fig. 5B), and toggle the bonding boxes of the objects. Also, a “Display Settings Bar” is available at the bottom of the screen, as shown in Fig. 6. It allows a user to set the global illumination constants (see Eq. 2), as well as the material reflection parameters for each type of object (Bundle or Tractography, Mesh, MRI).

### Object visualization

**Fig. 7:**
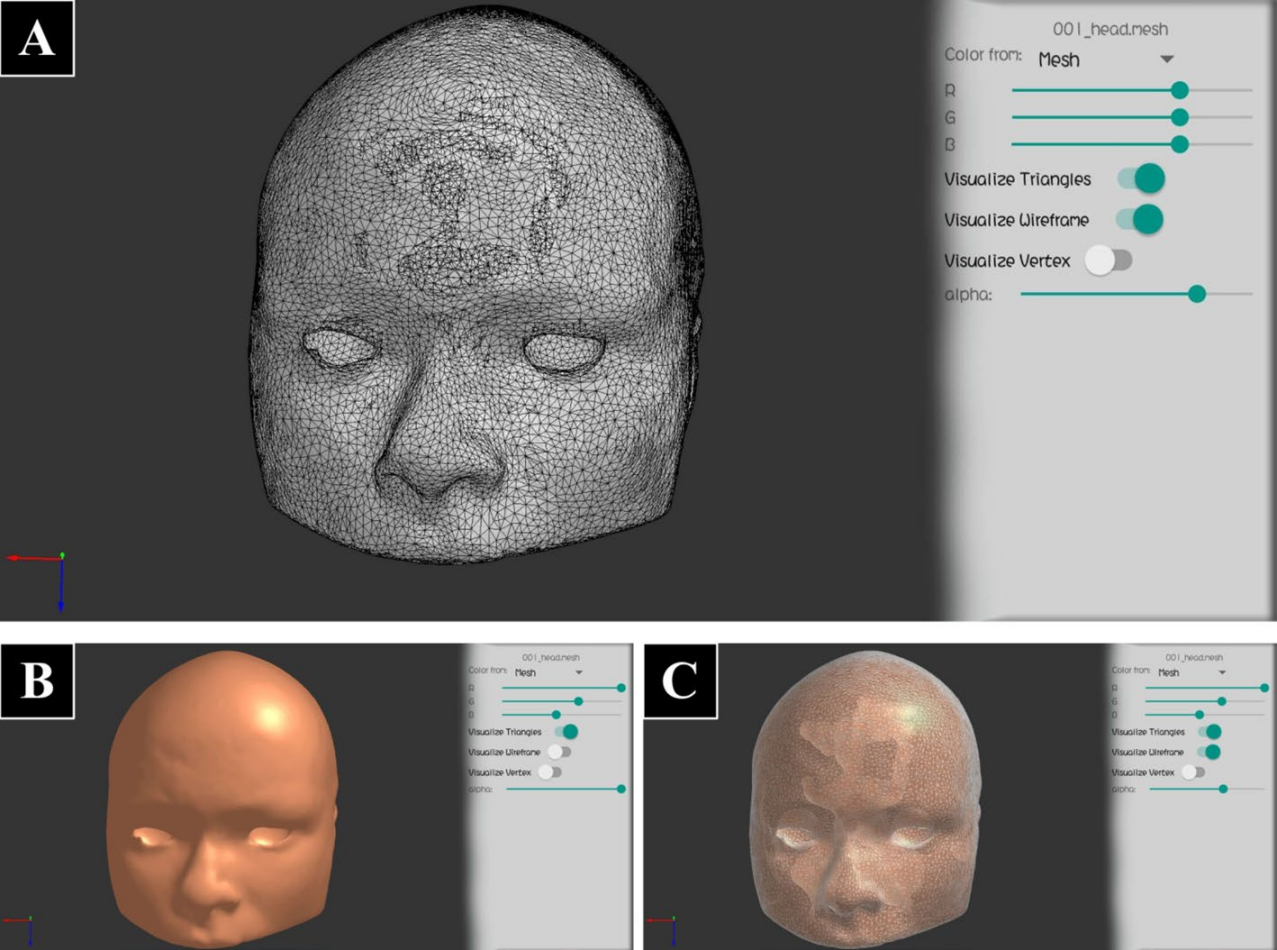
A mesh object of a head displayed with different visualization options (Dataset II). **A** Triangles and wireframe visible, with default colors, and high alpha for triangles (almost opaque). **B** Only triangles visible in copper color, with alpha=1.0 (totally opaque). **C** Triangles (in copper) and wireframe (in white) visible, with alpha=0.7 (triangles semi-transparent)

**Fig. 8:**
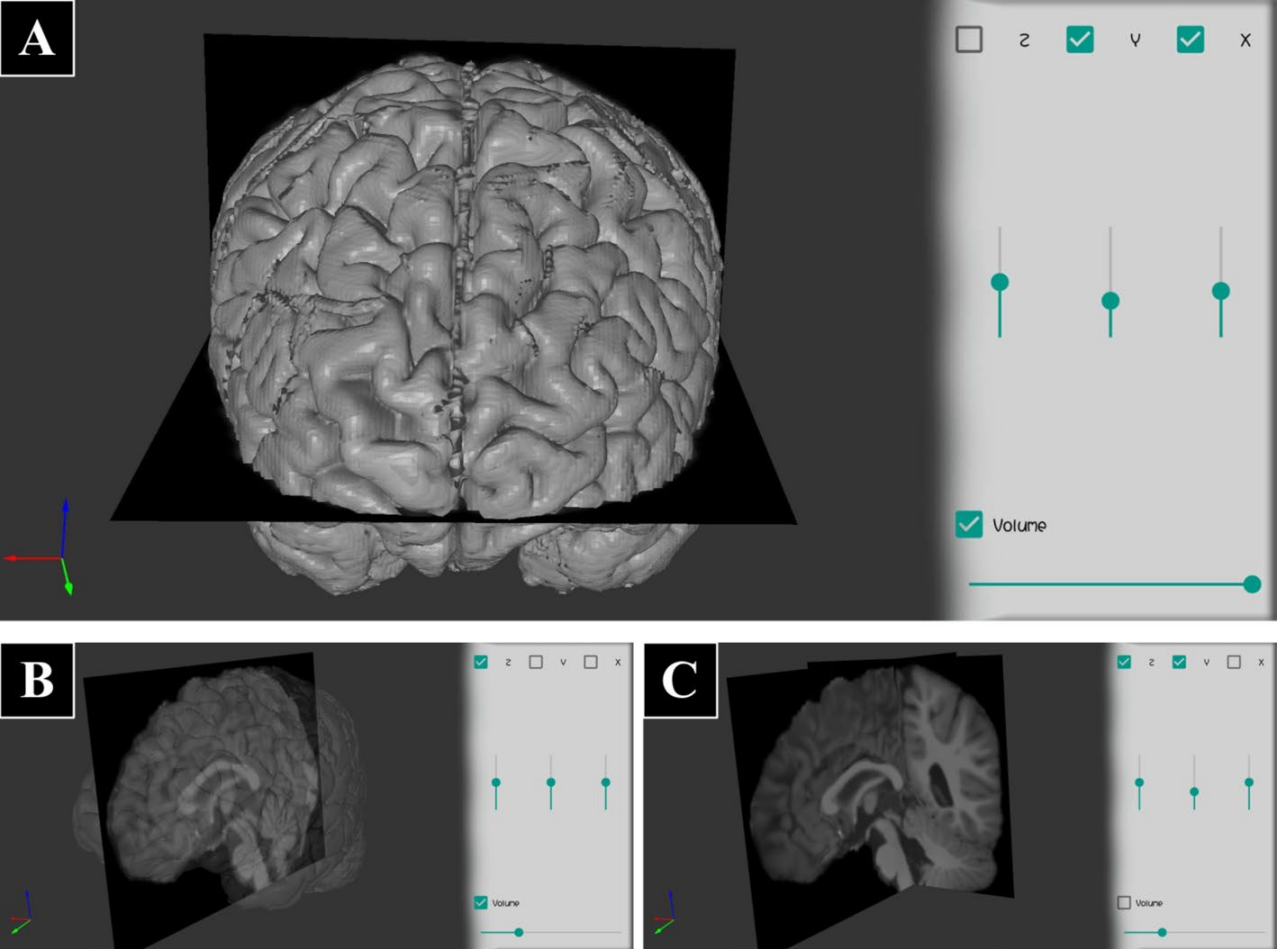
A brain MRI volume (without skull) displayed with different visualization options (Dataset IV). **A** Two slices, in axes “*X*” and “*Y*” of the volume, and a volume rendering of the image with maximum alpha, visualizing the cortical surface. **B** Only “*Z*” axis slice, and the volume rendering with alpha = 0.3 (semi-transparent). **C** Only two slices displayed, in axes “*Y*” and “*Z*”

**Fig. 9:**
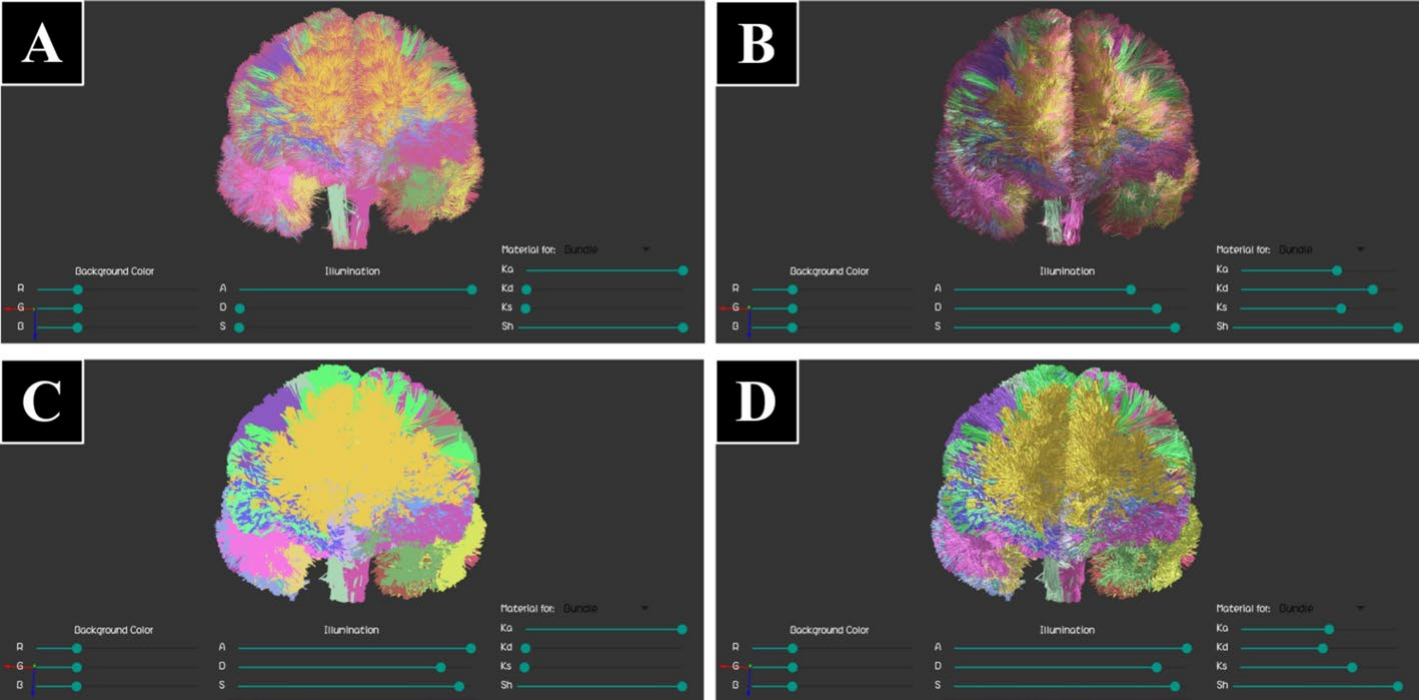
A tractography dataset with a group of segmented bundles (Dataset II), displayed with different visualization and illumination settings. The “Display Settings Bar” is also displayed at the bottom of the screen. **A** Line display with only ambient illumination ($A=1$, $D=0$, $S=0$). **B** Line rendering with high ambient, diffuse and specular illumination ($A=0.8$, $D=0.9$, $S=14$). **C** Cylinder rendering with a non-zero material reflection coefficient for ambient illumination ($Ka=1$, $Kd=0$, $Ks=0$), resulting in only an ambient light component. **D** Cylinder display with high ambient, diffuse and specular illumination ($A=1$, $D=0.9$, $S=0.95$)

**Fig. 10:**
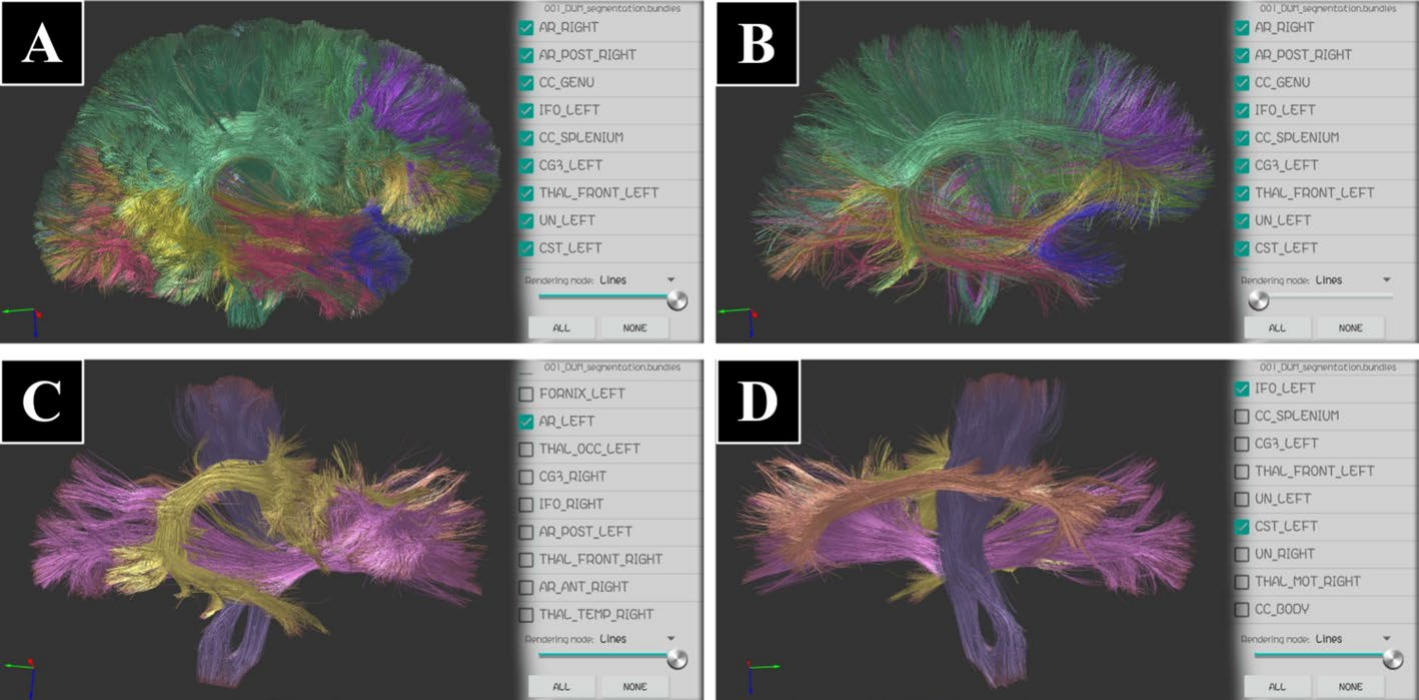
The tractography dataset with a group of segmented bundles (Dataset II), displayed with different percentages of fibers. **A** 100%, 204,052 fibers (all the bundles selected). **B** 10%, only 20,405 fibers selected. **C, D** 100% of the fibers for selected bundles of the left hemisphere (arcuate fasciculus, corticospinal tract, inferior fronto-occipital fasciculus and cingulum), sagittal lateral (**C**) and internal (**D**) views

Once an object is loaded, it can be selected in the “Object Settings Bar” (see Fig. 5B), to manipulate its display settings. The settings are specific to the three object types: Mesh, MRI and Tractography. Figure 7 shows different display options for a Mesh object, including different colors for the triangles and wireframe, and the setting of alpha value (transparency). Figure 8 shows examples of a visualization for MRI data using volume rendering and slices. This kind of object can be any 3D volume in NIfTI format. Figure 9 shows different display options for Tractography type, showing the effect of only ambient light component, compared to all the illumination components, and using lines or cylinder rendering. Additionally, Fig. 10 shows the option of fiber sampling and bundle selection.

**Fig. 11:**
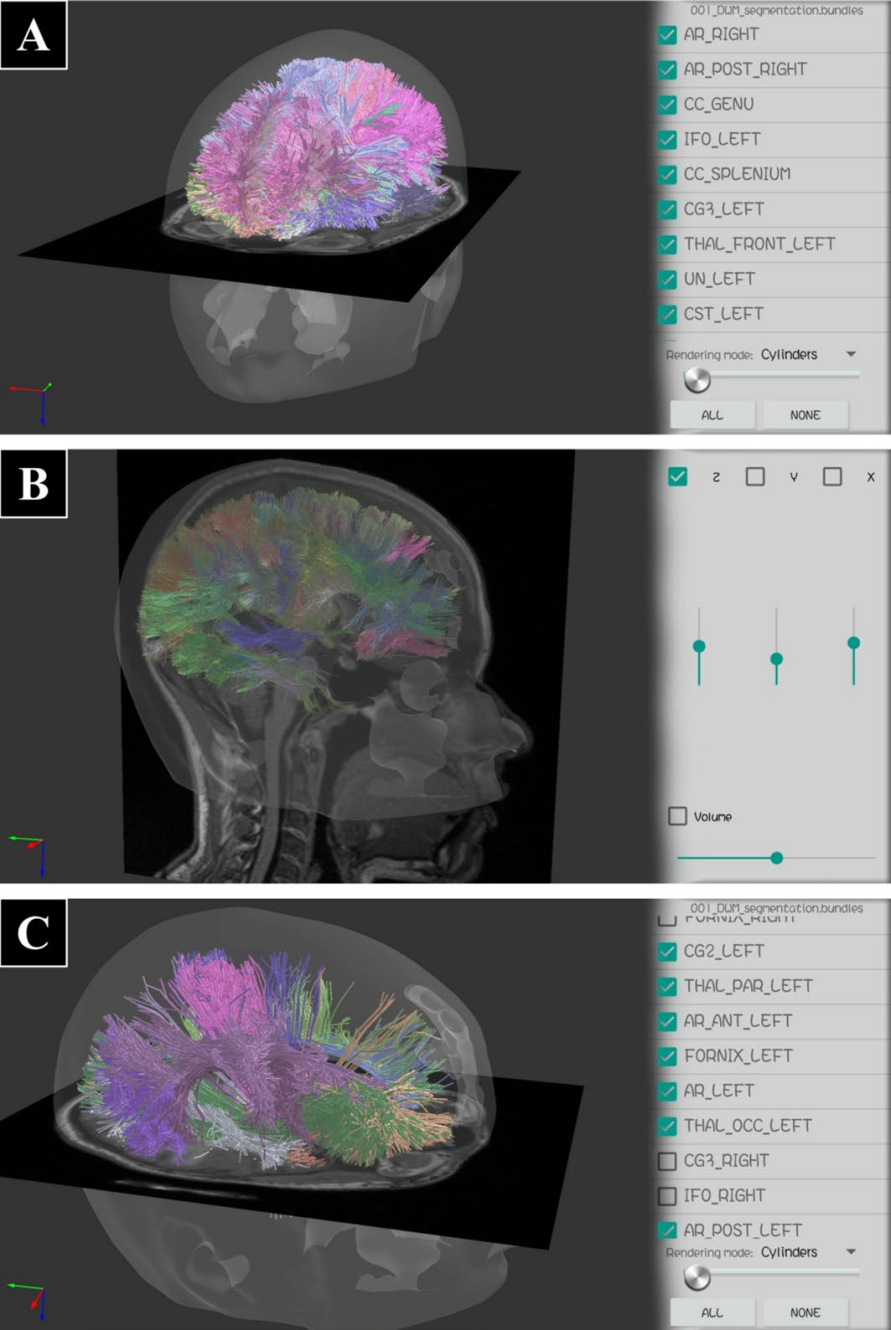
Example of brain data visualization of a healthy subject, consisting of a head mesh (semi-transparent), a brain MRI and a tractography dataset with labeled bundles (1% of the fibers displayed) (Dataset II). **A** An axial slice of the volume, all the bundles with cylinders, and the head mesh. **B** A sagittal slice, all the bundles with lines, and the head mesh. **C** An axial slice, some selected bundles with cylinders, and the head mesh

**Fig. 12:**
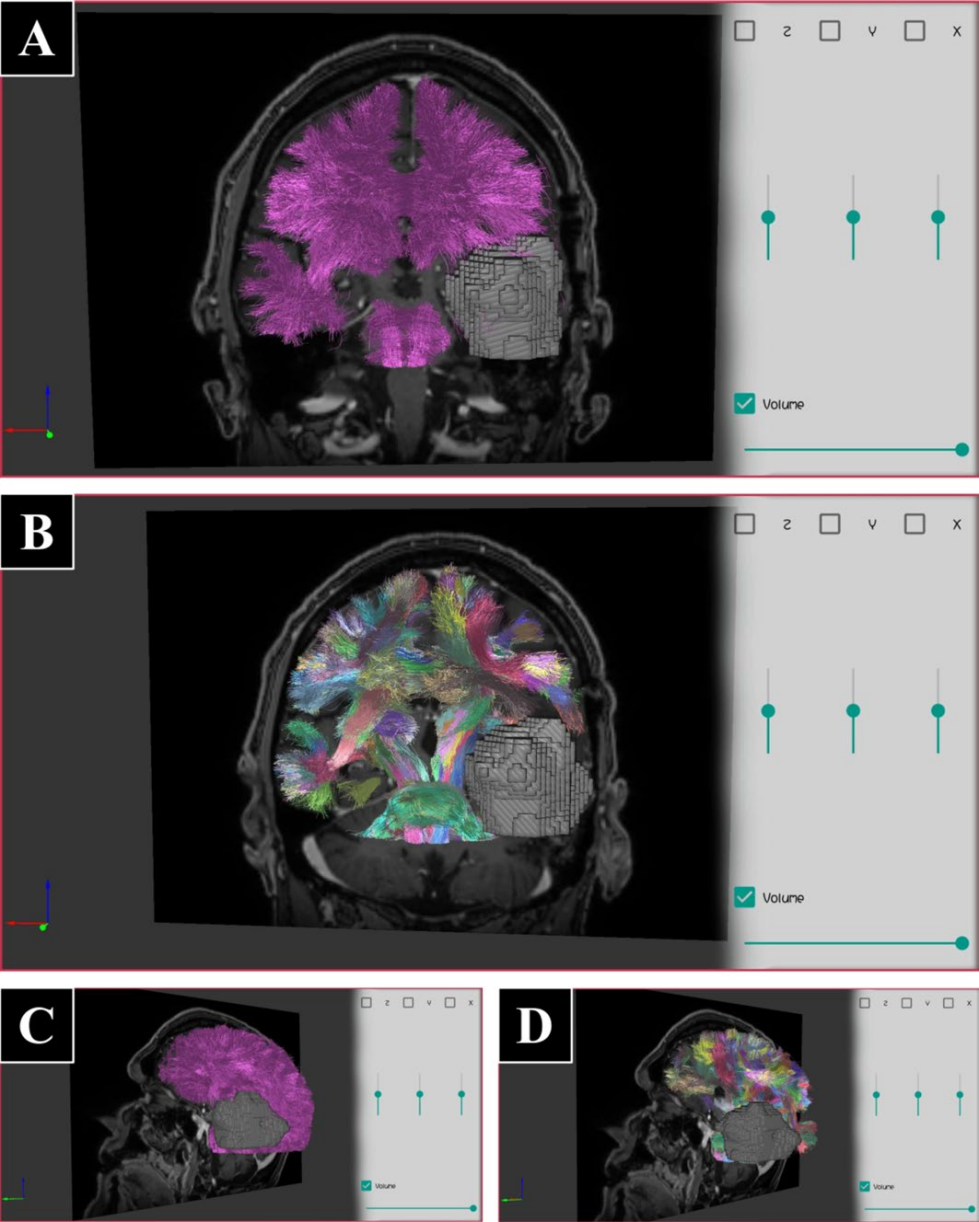
Example of brain data of a patient with a tumor, consisting of a brain MRI and a whole-brain tractography dataset with 71,361 fibers (Dataset III). Also, the dataset contains a brain mask of the segmented tumor which is displayed using volume rendering (VR), with maximum alpha, and a tractography dataset containing the 314 larger fiber clusters. **A** A coronal slice of the brain image, the whole-brain tractography displayed in purple, and the VR of the tumor. **B** A coronal slice of the brain, the 314 clusters displayed with random colors, and the VR of the tumor. **C, D** The same as **A** and **B**, respectively, but with a sagittal brain slice (sagittal oblique view)

**Fig. 13:**
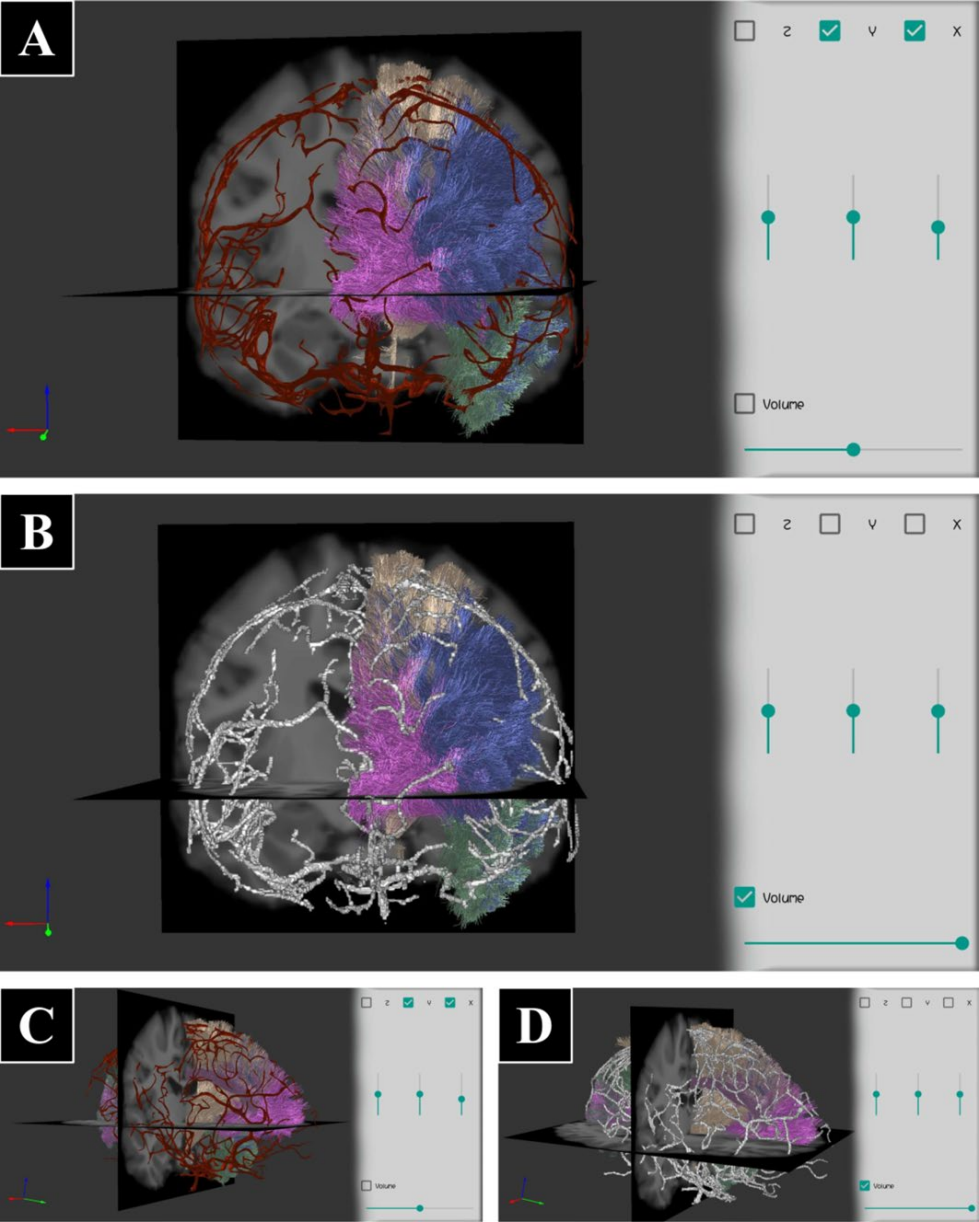
Example of brain data, including a brain MRI (skull stripped), a tractography dataset with labeled bundles, a mask of segmented arteries from MRI angiography and an arteries mesh (Dataset IV). **A** Coronal and axial slices of the brain image, the tractography displayed with random colors, and the arteries mesh in red. **B** The same slice and tractography display as in **A**, but with a volume rendering of the arteries mask, instead of the mesh (with maximum alpha). **C, D** The same as **A** and **B**, respectively, but from a sagittal oblique view

Finally, Figs. 11, 12 and 13 illustrate three case studies providing different types of visualizations supported by ABrainVis, for Datasets II, III and IV, respectively.

For all the figures, we provide dataset information and display options details in the caption of each figure.

### Performance results

This section describes the experiments performed to evaluate the visualization time, using the cell phones and tablet devices listed in Table 3, for the tractography of Dataset II (204,052 fibers).

**Fig. 14:**
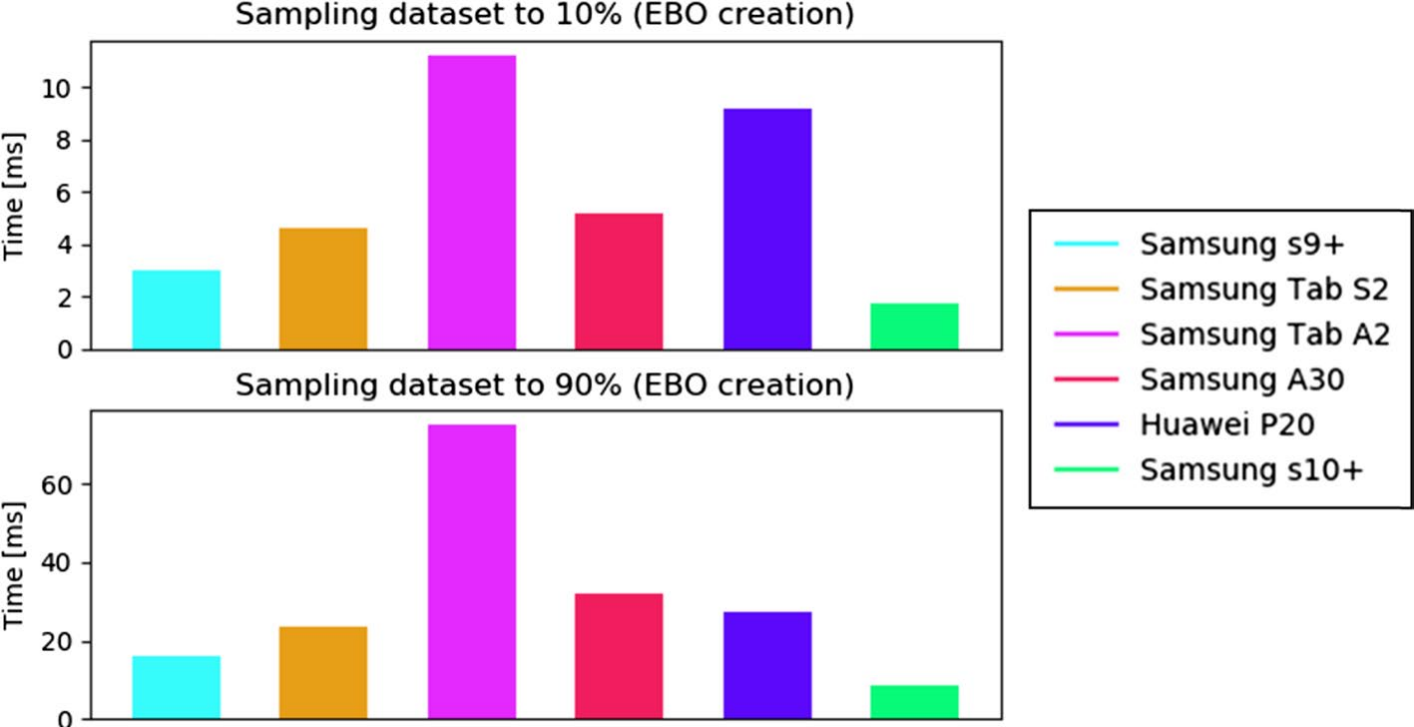
Time required for the creation of the fiber sampling data (EBO creation), for two different fiber sampling percentages (10% and 90%), measured in the six devices listed in Table 3. The tractography used has 204,052 fibers (Dataset II)

The first experiment evaluates the time required for the creation of the fiber sampling data (EBO creation), for two different fiber sampling percentages (10% and 90%). Figure 14 shows such results. As expected the creation and EBO binding time increases when the percentage of fibers considered grows, but it is still reasonably low, with a mean of about 20 seconds for 90 % of the fibers (184,068 fibers) for the medium- to high-quality devices (all the devices, excepting Tablet Samsung Tab S2, which is quite old).

**Table 3:** Mobile devices main features

		Features			
	ID	Model	CPU	GPU	RAM
Cell phones	C1	Samsung S9+	Snapdragon 845	Adreno 630	6 GB
	C2	Samsung S10+	Exynos 9820	Adreno 640	8 GB
	C3	Huawei P20	Kirin 970	Mali-G72	4 GB
	C4	Samsung A30	Exynos 7904	Mali-G71	3 GB
Tablets	T1	Samsung Tab S2	Exynos 5433	Adreno 510	3 GB
	T2	Samsung Tab A (2016)	Exynos 7870 Octa	Mali-T830	3 GB

**Fig. 15:**
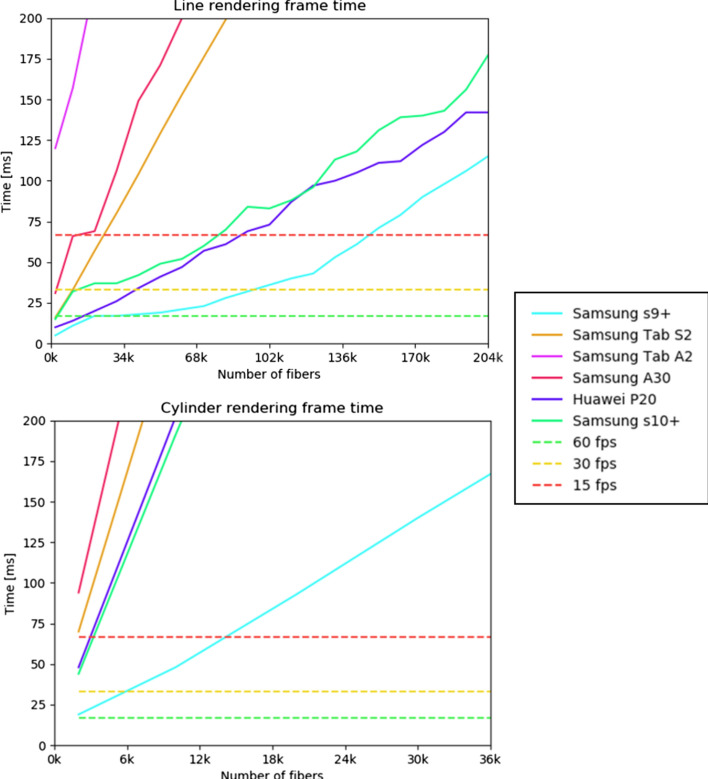
Frames per second (fps) obtained using different percentage of fibers for line and cylinder rendering, when displaying the tractography of Dataset II (204,052 fibers), measured in the six devices listed in Table 3

The second experiment evaluates the frames per second (fps) achieved, using different percentage of fibers for line and cylinder rendering (see Fig. 15). The fps decreases with the number of fibers displayed, where 60 fps is the optimal and 15 fps is the minimum to have an interactive display. As can be seen for line rendering, about 145,000 fibers can be displayed with the device of higher performance, and a mean of about 70,000 fibers can be displayed, considering all the devices, excepting the Tablet Samsung Tab S2. In the case of cylinders, the quantity of fibers that can be displayed decreases to a maximum of 13,000 fibers and a mean of about 5000 fibers. In any case, note that cylinder display is more useful when displaying few fibers.

## Conclusions

We present a mobile application for Android devices for the visualization of imaging data. This tool is very versatile since it supports three types of data, namely, labeled tractography datasets, 3D images and meshes. It offers numerous display options, uncommon for applications of this type. Given the massive use of mobile devices, we believe it may be of great interest to researchers, clinicians, and educators. As far as we know, this is the most complete application of this type that exists. We offer it as open source, so that the community can contribute to its development and improvement.

Future work will be focused on the addition of more visualization options. For example, the support of an affine transformation data type, that could be applied to any object, or the addition of different color maps for 3D volumes. Also, it would be useful to support a mesh label data type, for applying different colors to the mesh vertices, and be able to color different regions over a mesh. Regarding the volume rendering tool, we plan to add the support of a transfer function, for associating a different alpha value to each voxel intensity. Finally, the support of a configuration file would provide the capability to store and load display options for a group of objects, enabling a fastest display of a dataset.

## Supplementary Information

**Additional file 1: Table S1.** Neuroimaging visualization tools.

**Additional file 2.** Demo video file.

## Data Availability

The source code of the ABrainVis is available on a repository of github. Project name: ABrainVis. Project home page: https://github.com/Cocobio/aBrainVis. Operating system for development: Windows 10. Programming language: Java, GL Shader Language (GLSL). Other requirements for development: Android Studio 3.5 or higher, OpenGL ES 3.2, JRE 1.8.0 or higher. Mobile operating system: Android 6.0 or higher. License: GNU General Public License v3.0 (non-commercial use).

## Abbreviations

CPU: Central processing unit
CT: Computer tomography
DAE: Digital asset exchange file format
dMRI: Diffusion magnetic resonance imaging
EBO: Element buffer object
EEG: Electroencephalography
FFClust: Fast fiber clustering
fps: Frames per second
FS: Fragment Shader
GIfTI: Geometry informatics technology initiative
GUI: Graphic user interface
GPU: Graphics processing unit
GS: Geometry shader
iOS: iPhone operating system
MRI: Magnetic resonance imaging
NIfTI: Neuroimaging informatics technology initiative
OBJ: Wavefront 3D object file
OpenGL: Open graphics library
OpenGL ES: OpenGL for embedded systems
PET: Positron emission tomography
RAM: Random access memory
STL: Stereolithography file
VBO: Vertex buffer object
VR: Volume rendering
VS: Vertex shader
2D: 2-Dimensional
3D: 3-Dimensional
