# Supplementary material for: ABrainVis: an android brain image visualization tool
Source: Biomed Eng Online. 2021 Jul 29;20:72. doi: 10.1186/s12938-021-00909-0 (PMC8323223; doi:10.1186/s12938-021-00909-0)
Supplement: Supplementary file 1 — Additional file 1: Table S1. Neuroimaging visualization tools. [file 12938_2021_909_MOESM1_ESM.pdf]

Supplementary Material
ABrainVis: An android brain image visualization tool

Ignacio Osorio, Miguel Guevara, Danilo Bonometti, Diego Carrasco,
Maxime Descoteaux, Cyril Poupon, Jean-François Mangin,
Cecilia Hernández, and Pamela Guevara

Comparison between neuroimaging visualization tools

Table 1 presents the main features of the available neuroimaging visualization tools discussed in the Background section of the manuscript. However, this is not an exhaustive list, as the selected tools are mainly mobile applications.

For each tool, we provide information about the supported platform including the operating system (MacOS, Windows, Linux) when found in corresponding documentation, or a more general description, such as a Web or a Personal Computer (PC) application. In addition, we include information about the types of input data that each tool supports. To reduce the space in the table we use the following acronyms:

- dMRI_v: diffusion MRI volume
- MRI_v: MRI volume
- CT: Computer Tomography slices
- MRI_s: MRI slices
- Trac: tractography data
- f_act: functional activations
- fMRI ConnNet: functional MRI Connectivity Networks
- US: ultrasound images

In the case the supported data types are mentioned, we also include formats, such as DICOM, NiFTI, GiFTI, trk, bundles, and nrrd/nhdr.

Next, we include another column in the table with the ability of the tool to operate with internal (I) or external (E) data. Operation with internal data means that the tool uses predefined data and users are restricted to use only that data. On the other hand, operation with external data means that the tool allows users to load and process their own data.

In the next column, we include the visualization features of each tool. Again, to reduce space we use the following acronyms:

- 2Ddisp: 2D image display
- sNav: slice navigation
- rot: rotating
- pan: panning
- zoom: zooming
- trans: transparency

- 3Drend: 3D rendering
- sup: superimposing structures
- ROI: ROI display

The final column provides the tool availability (Av) is noted by an F, if it is freely available or a P if it requires a payment.

Furthermore, the web page of each tool (if available) is referenced with a number as a superindex, after the name of each application. The list of web pages is included here:

- [0] http://dmri.slicer.org/
- [1] http://bit.ly/1kEJH6q
- [2] https://brainbox.pasteur.fr/
- [3] http://www.tractometer.org/fiberweb/
- [4] https://tissuestack.org/
- [5] https://www.brainvoyager.com/Mobile/BrainTutor3D_Android.html
- [6] https://play.google.com/store/apps/details?id=com.appsclinical.atlasofmribrainanatomydraft&hl=es&gl=US
- [7] https://apps.apple.com/us/app/brain-mri-atlas/id431556580
- [8] https://play.google.com/store/apps/details?id=com.russ.fiber_visualizer&hl=en_US&gl=US
- [9] https://play.google.com/store/apps/details?id=mdtoolkit.mriviewer
- [10] https://play.google.com/store/apps/details?id=com.andromo.dev658544.app1004140&hl=es_419&gl=US
- [11] https://play.google.com/store/apps/details?id=com.radrevision.frcr1anatomyrevisionapp&hl=es&gl=US
- [12] https://play.google.com/store/apps/details?id=com.andromo.dev658544.app1004162&hl=fr_CA&gl=US
- [13] https://play.google.com/store/apps/details?id=org.homphysiology.neuroslice&hl=es_BO
- [14] https://play.google.com/store/apps/details?id=com.drb.brains&hl=en&gl=US
- [15] https://play.google.com/store/apps/details?id=com.aribraindev.iBraiNEEG2&hl=en_US&gl=US
- [16] https://play.google.com/store/apps/details?id=org.mes&hl=es&gl=US
- [17] https://play.google.com/store/apps/details?id=com.imaios.imaiosdicomviewer&hl=es&gl=US

Table 1: Neuroimaging visualization tools

Visualizator	Platform	Type of input data	Input	Visualization features	Other features (besides visualization)	Av
SlicerDMRI [1] [0]	Mac Windows Linux	dMRI_v (DICOM and nrrd/nhdr)	E	2Ddisp	Calculate load and save tractography, Compute diffusion-based scalar maps, Edit and register multimodal data	F
Wang J. et al [2]	PC	MRI_v (DICOM/NiFTI)	E	sNav, rot	Image proccesing for obtaining the brain mesh from T1 MRI	F
brainR [3][1]	Web	3D and 4D data (longitudinal)	E	zoom (click), pan (click) rot (click), trans, Coloring of brain substructures	Addition/removal of surfaces to see changes longitudinally	F
BrainBox [4][2]	Web	MRI_v	E	sNav (slider), rot (3D render), 3Drend	Measurements (distances), ROI drawing/annotations	F
FiberWeb [5][3]	Web	MRI_v (NiFTI) and Tract	E	zoom (click), pan (click), rot (click)	Fiber virtual dissection, Real time fiber tractography, Saving parameteres/results	F
Tissue Stack [6] [4]	Web	3D volumes (MINC/NiFTI)	E	pan (click), tiling of images	None	F
Brain Tutor [7] [5]	Android iOS	MRI_v Mesh Tract and f_act	I	sNav (swipe), zoom (touch), pan (touch), rot (touch), sup, Fiber color change	Touch structure for labeling display, Button for display structure information	F/P
Atlas of Brain Anatomy [8] [6]	Android	MRI_s	I	zoom (touch), ROI	Labels about the lobes, brainstems and cerebellum, basal ganglia and thalamus, white matter, CSF spaces and vessels, Links to online brain structure information (urls).	P
Brain MRI atlas [9] [7]	iOS	MRI_v	I	sNav (slider), Swipe between images	Labeled brain structures	F
NeuroNavigator [10] [8]	Android	MRI_v Mesh Tract and f_act	I	sNav (swipe), zoom (touch), rot (touch), trans, sup, fiber coloring (4 modes by button)	Fiber length filter and fiber sampling, Fiber virtual dissection (through ROIs), ROI touching for label display	F
MRI viewer [11] [9]	Android	MRI_v	I	sNav (swipe), zoom (touch and button)	None	F
CT Scan Cross Sectional Anatomy for Imaging Pros [12] [10]	Android	CT and MRI_s	I	2Ddisp	More than 1,500 images, Hand annotated structures (over the images), Description, location and function of the anatomy	F
Radiological Anatomy for FRCR1 [13] [11]	Android	X-ray CT and MRI_s US Fluoroscopy	I	2Ddisp	500 images with one structure labelled per image, Textual information of the structure	P
Imaging Brain, Skull, Craniocervical Vasculature [14] [12]	Android	CT and MRI_s dMRI_s	I	2Ddisp	Show images with variants of brain anatomy, skull, craniocervical vasculature	F
NeuroSlice [15] [13]	Android	MRI_s	I	ROI, Image seletion	40 images with labeled structures, Touch structure for information display	F
Myelination Brain [16] [14]	Android	MRI_v	I	sNav (scroll)	Information of brains from 0 to 24 months of age	P
Dogan I. et all	iOS	Photos of lesions	E	Augmented reality (superposing lesion images over the background)	None	F
iBraiN/iBrainNEEG [17] [15]	Android	Mesh fMRI ConnNet	I	zoom, pan, rot (button)	Touch region/electrode for label showing	F
mRay [18] [16]	iOS Android Windows	DICOM	E	2Ddisp	Text annotations and distance measurement, Managing of a DICOM image database, Sharing key images, audio and text messages	F
IMAIOS Dicom Viewer [19] [17]	iOS Android Web	DICOM	E	zoom, pan, rot (touch), Adjust brightness and contrast	Distance measurement	F
ABrainVis	Android	MRI_v (NiFTI), Mesh (GiFTI/mesh) and Tract (trk/bundles)	E	zoom (touch), pan (touch), rot (touch), trans, sNav 3Drend, sup, Illumination settings	White matter bundle atlases, Example of whole-brain fiber clusters, fiber sampling	F

The following figures show examples of visualization for the three most similar applications to ABrainVis (*BrainTutor*, *NeuroNavigator* and *FiberWeb*). Only ABrainVis and FiberWeb support external data. Figure 1 presents an example of visualization with *FiberWeb*. Although different tracts are loaded, it is not possible to visualize them with different colors. Furthermore, the head mesh could not be loaded since mesh files are not supported.

Figures 2 and 3 show visualization examples with *BrainTutor* and *NeuroNavigator*, respectively.

All these tools provide visualizations that are of interest for the research area, however *BrainTutor* and *NeuroNavigator* are restricted to predefined data. FiberWeb and ABrainVis are more flexible, enabling users to load their own data for visualization. In addition, ABrainVis has the advantage over FiberWeb of allowing users to incorporate mesh data, which provides users the analysis of additional visualization context such as cortical and subcortical structures and arteries.

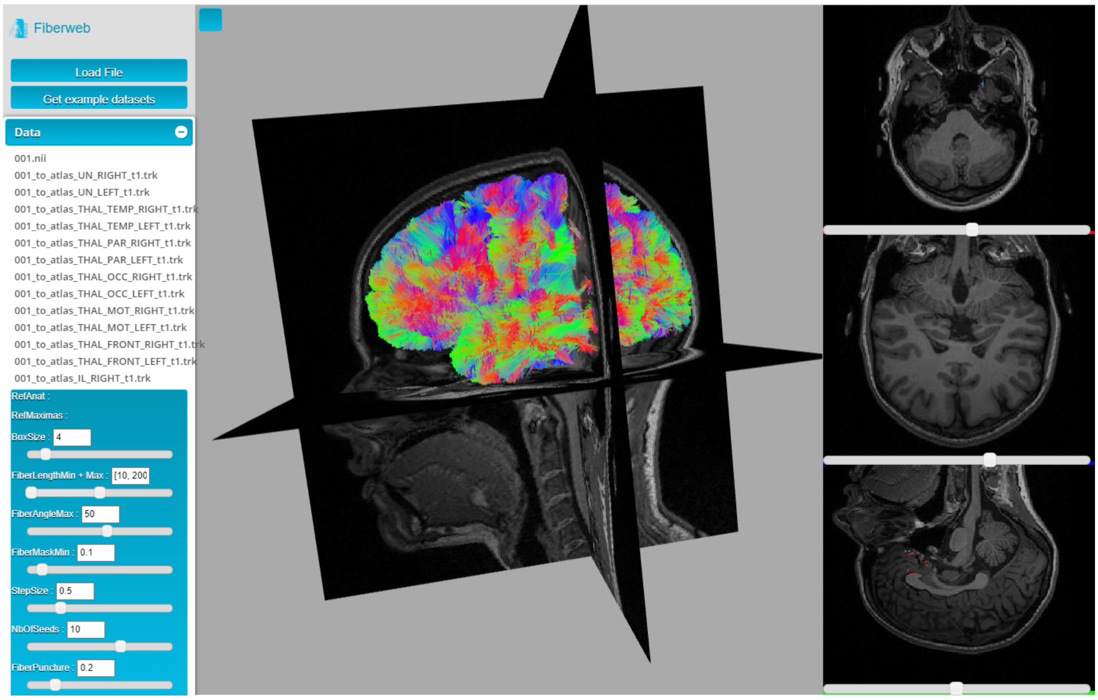

Figure 1: Example of visualization generated by FiberWeb for *Dataset II*, (Figure 11 of the manuscript), consisting of a brain MRI and tractography datasets for 36 segmented fascicles.

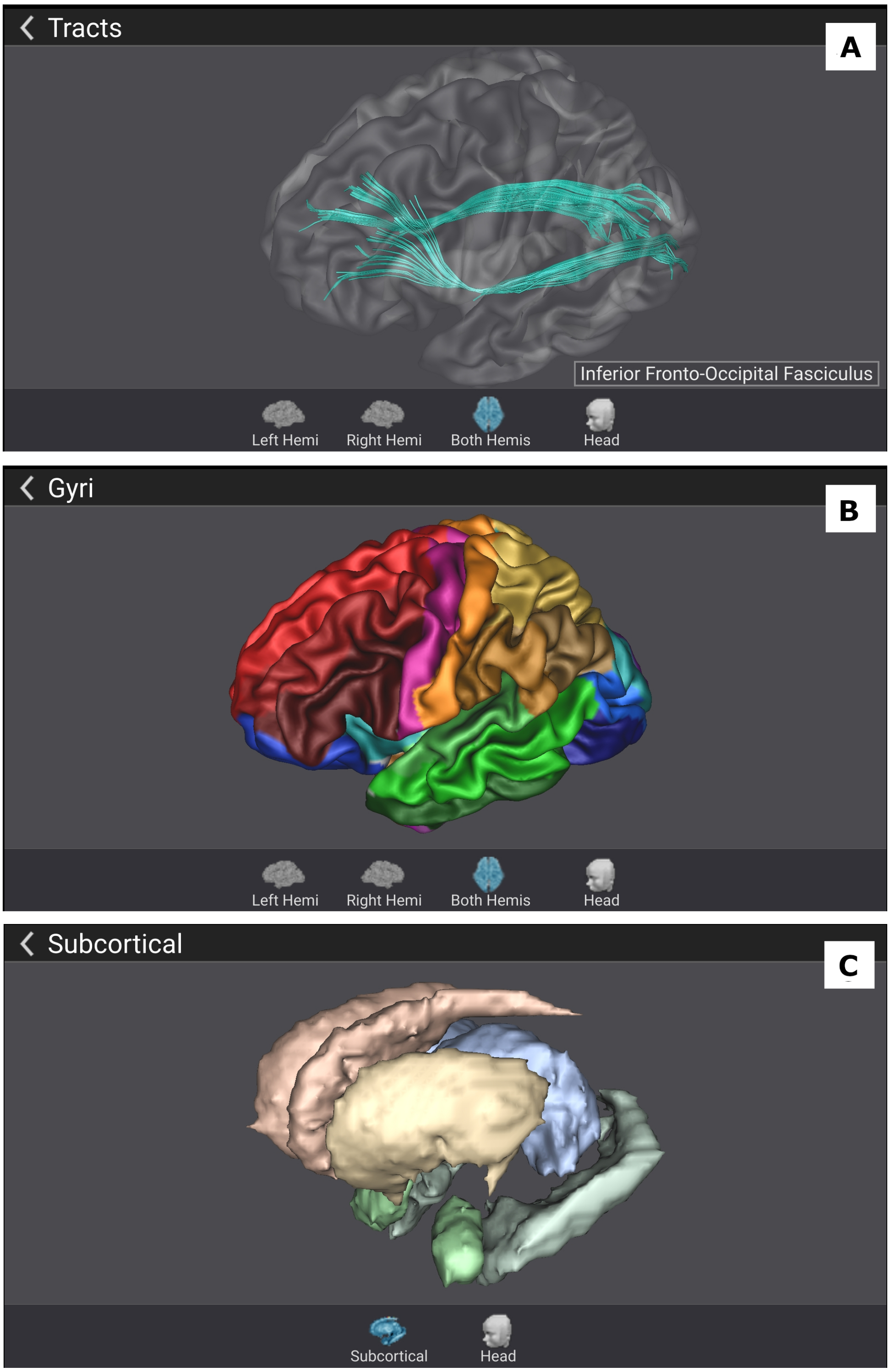

Figure 2: Example of visualization generated by BrainTutor (preset data only). A) Semi-transparent cortical meshes and a fiber tract for both hemispheres (only one fiber tract can be displayed at a time). B) Cortical meshes with gyri visualized in different colors. C) Meshes for subcortical structures in different colors.

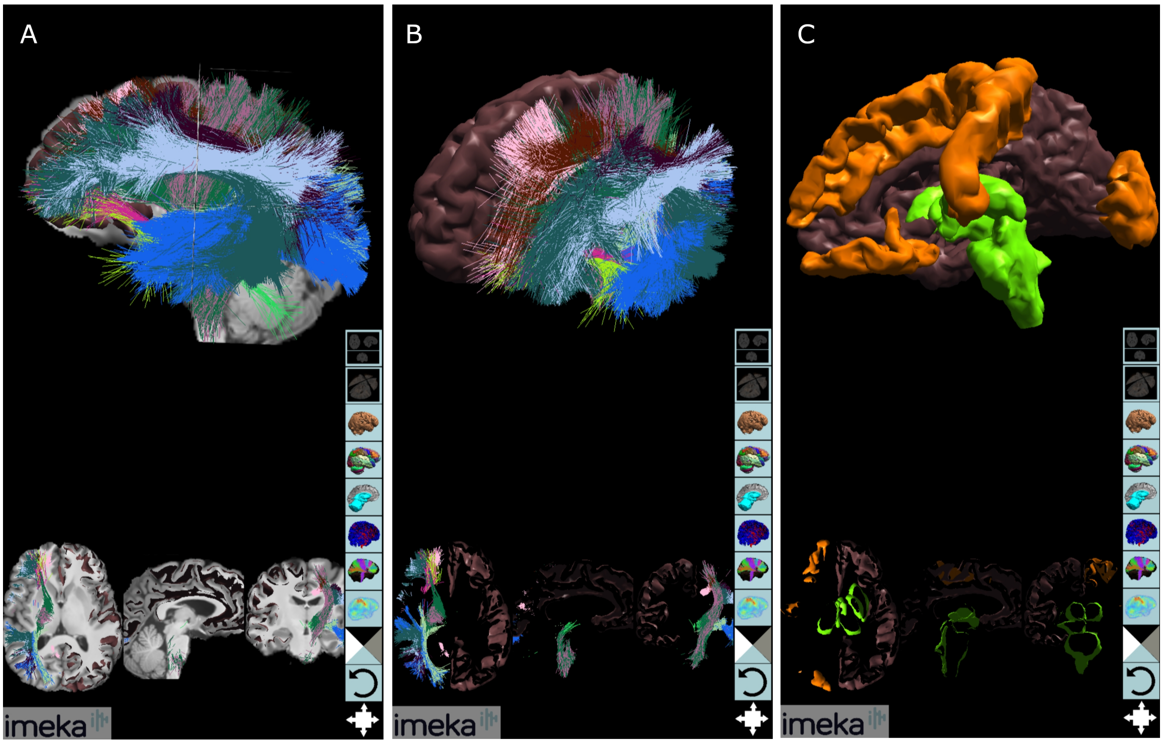

Figure 3: Example of visualization generated by NeuroNavigator (preset data only). A) Slices for a skull-stripped brain image and a selection of left white matter tracts (displayed in different colors). B) The same white matter tracts as in A), but with a right cortical mesh instead of the brain image (in brown). C) The right cortical mesh (in brown) and a random selection of gyri (in orange) and subcortical structures (in green).

References

[1] Norton, I., Essayed, W.I., Zhang, F., Pujol, S., Yarmarkovich, A., Golby, A.J., Kindlmann, G., Wassermann, D., Estepar, R.S.J., Rathi, Y., *et al.*: SlicerDMRI: Open source diffusion MRI software for brain cancer research. Cancer Research **77**(21), 101–103 (2017)

[2] Wang, J., Sun, Z., Ji, H., Zhang, X., Wang, T., Shen, Y.: A fast 3D brain extraction and visualization framework using active contour and modern OpenGL pipelines. IEEE Access **7**, 156097–156109 (2019)

[3] Muschelli, J., Sweeney, E., Crainiceanu, C.: BrainR: interactive 3 and 4D images of high resolution neuroimage data. The R journal **6**(1), 41 (2014)

[4] Heuer, K., Ghosh, S., Sterling, A.R., Toro, R.: Open neuroimaging laboratory. Research Ideas and Outcomes **2**, 9113 (2016)

[5] Ledoux, L.-P., Morency, F.C., Cousineau, M., Houde, J.-C., Whittingstall, K., Descoteaux, M.: Fiberweb: diffusion visualization and processing in the browser. Frontiers in Neuroinformatics **11**, 54 (2017)

[6] Lin, M.K., Nicolini, O., Waxenegger, H., Galloway, G., Ullmann, J., Janke, A.: Interpretation of medical imaging data with a mobile application: a mobile digital imaging processing environment. Frontiers in Neurology **4**, 85 (2013)

[7] Brain Tutor: Android. `https://www.brainvoyager.com/Mobile/BrainTutor3D_Android.html`. [Online; accessed 28-December-2020] (2020)

[8] Atlas of MRI Brain Anatomy. `https://play.google.com/store/apps/details?id=com.appsclinical.atlasofmribrainanatomydraft`. [Online; accessed 28-December-2020] (2020)

[9] Minkowitz, S.: Review of "brain mri atlas" app for the ipad. Journal of digital imaging **28**(6), 633 (2015)

[10] NeuroNavigator. `https://play.google.com/store/apps/details?id=com.russ.fiber_visualizer&hl=es_CL&gl=US`. [Online; accessed 28-December-2020] (2020)

[11] MRI Viewer. `https://play.google.com/store/apps/details?id=mdtoolkit.mriviewer`. [Online; accessed 28-December-2020] (2020)

[12] CT Scan Cross Sectional Anatomy for Imaging Pros. `https://play.google.com/store/apps/details?id=com.andromo.dev658544.app1004140`. [Online; accessed 28-December-2020] (2020)

[13] Radiological Anatomy For FRCR1. `https://play.google.com/store/apps/details?id=com.radrevision.frcr1anatomyrevisionapp`. [Online; accessed 28-December-2020] (2020)

[14] Imaging Brain, Skull & Craniocervical Vasculature. `https://play.google.com/store/apps/details?id=com.andromo.dev658544.app1004162`. [Online; accessed 28-December-2020] (2020)

[15] NeuroSlice. `https://play.google.com/store/apps/details?id=org.homphysiology.neuroslice`. [Online; accessed 28-December-2020] (2020)

[16] Myelination Brain. `https://play.google.com/store/apps/details?id=com.drb.brains&hl=es_CL&gl=US`. [Online; accessed 28-December-2020] (2020)

[17] Rojas, G.M., Fuentes, J.A., Gálvez, M.: Mobile device applications for the visualization of functional connectivity networks and EEG electrodes: iBraiN and iBraiNEEG. Frontiers in Neuroinformatics **10**, 40 (2016)

[18] mRay. `https://play.google.com/store/apps/details?id=org.mes`. [Online; accessed 28-December-2020] (2020)

[19] IMAIOS Dicom Viewer. `https://play.google.com/store/apps/details?id=com.imaios.imaiosdicomviewer`. [Online; accessed 28-December-2020] (2020)
